# Prevalence of Carbapenemase Production Among Klebsiella and Escherichia coli Isolated From Urinary Tract Infections

**DOI:** 10.7759/cureus.70918

**Published:** 2024-10-06

**Authors:** Kumuda Arumugam, Geeta S Karande, Satish R Patil

**Affiliations:** 1 Department of Microbiology, Krishna Vishwa Vidyapeeth (Deemed to be University), Satara, IND

**Keywords:** antimicrobial susceptibility, carbapenemase, carbapenem-resistant enterobacteriaceae (cre), e.coli, healthcare-associated infections, klebsiella, mcim, mht, phenotypic methods, urinary tract infection

## Abstract

Background and aim

Urinary tract infections represent a substantial portion of healthcare-associated infections due to *E. coli* and *Klebsiella*. Carbapenems are broad-spectrum antibiotics considered highly effective in treating infections caused by multidrug-resistant bacteria. Carbapenem-resistant *Enterobacteriaceae (*CRE), including carbapenem-producing *E. coli* and *Klebsiella* isolates, have become a major concern as they limit treatment options. The study aims to determine the prevalence of carbapenemase-producing *E. coli* and *Klebsiella* while also comparing the effectiveness of two detection methods, namely the modified carbapenem inactivation method (mCIM) and modified Hodge test (MHT).

Materials and methods

A cross-sectional study was conducted from July 2022 to June 2023 in a tertiary care hospital, in Karad, Satara, India. Three hundred urinary isolates of *E. coli* (150) and *Klebsiella* (150) were studied. These isolates were tested for antimicrobial susceptibility testing. Two phenotypic methods, the modified carbapenem inactivation method (mCIM) and the modified Hodge test (MHT), were used to study carbapenemase production.

Results

Out of three hundred isolates, carbapenemase production was detected in 72 isolates (24%) by the modified Hodge test (MHT) and in 111 isolates (37%) by the modified carbapenem inactivation method (mCIM). Among the MHT-positive isolates, 46 (63.8%) were identified as *Klebsiella* and 26 (36.1%) as *E. coli*. In contrast, of the mCIM-positive isolates, 68 (61.2%) were *Klebsiella*, and 43 (38.7%) were *E. coli*. A total of 41 *Klebsiella* (27.33%) and 25 *E. coli* (16.66%) isolates tested positive by both methods, highlighting variability in detection rates between the two methods.

Conclusion

This study observed MHT and mCIM to be accurate for the detection of carbapenemase among carbapenem-resistant isolates. However, the mCIM demonstrated greater sensitivity compared to the MHT.

## Introduction

Multi-drug-resistant (MDR) *Enterobacteriaceae* infections represent a serious concern to public health because of their high mortality rates. Carbapenem-resistant *Enterobacteriaceae* (CRE) are among the most important antibiotic-resistant bacteria, making them highly dangerous. The multidrug resistance rates in *Escherichia coli* and *Klebsiella* *pneumoniae* are reported to be 30% and 50%, respectively, by the Indian Council of Medical Research (ICMR) [1]. Classical symptoms like fever, dysuria, etc. are indicative of urinary tract infections (UTI), as is a urine culture that shows the growth of identified uropathogens above 100 cfu/ml to 105 cfu/ml [2]. *E. coli* is the most common etiologic agent of UTI [3], with *Klebsiella* coming in second.

The use of carbapenems in gram-negative bacilli infections that produce extended-spectrum beta-lactamase (ESBL) is increasing [4]. These are a class of beta-lactam antibiotics with broad antibacterial activity. They act by preventing the penicillin-binding proteins (PBPs), thereby inhibiting the synthesis of bacterial cell walls [5]. This disruption of cell wall production leads to bacterial cell lysis and death.

Carbapenem-resistant *Enterobacteriaceae* (CRE) are referred to as resistance to one or more of the carbapenems: meropenem, ertapenem, and imipenem [6]. Carbapenem resistance in *Enterobacteriaceae* is primarily due to carbapenemase enzyme production. In addition to carbapenemase enzyme production, other resistance mechanisms include the over-expression of efflux pumps by the bacteria, the absence of porins in the bacterial cell membrane, and reduced binding of carbapenems to penicillin-binding proteins [7].

These enzymes are encoded by various genotypes and can be spread among *Enterobacteriaceae* through transferable genetic material. Notable enzymes involved include Class A carbapenemase, such as *Klebsiella* *pneumoniae*. Carbapenemase (KPC) belongs to the serine beta-lactamase family; Class B carbapenemase, known as metallo-beta-lactamase (MBL), includes New Delhi metallo-beta-lactamase (NDM), Verona integron-encoded metallo-beta-lactamase (VIM), and imipenemase (IMP) and needs metal ions to function; and Class D carbapenemase, or oxacillinases, comprises various subgroups like OXA-48-like enzymes [8]. Understanding this carbapenemase classification is essential for combating antibiotic resistance and guiding treatment strategies against multidrug-resistant bacteria.

Carbapenem-resistant *Enterobacteriaceae* (CRE) pose a serious threat to global health due to their resistance to carbapenems, which are usually the last resort against multidrug-resistant bacterial infections. CRE infections, which are caused by *Klebsiella* *pneumoniae* and *Escherichia coli*, have been linked to 50% mortality rates, particularly in vulnerable populations like immunocompromised patients [9]. Low- and middle-income countries have a high rate of CRE due to ineffective infection control protocols, inadequate healthcare infrastructure, and restricted access to advanced diagnostic equipment. The World Health Organization (WHO) noted limited treatment options available for CRE, especially in underdeveloped regions, and its rapid spread as reasons for designating it as a major priority for worldwide research and antibiotic development. The emergence of CRE is further aided by overcrowded medical facilities, inadequate sanitation, and extensive antibiotic abuse [10]. The economic burden of CRE infections is substantial, burdening fragile healthcare systems with longer hospital stays, isolation needs, and costly last-line antibiotics. Addressing CRE necessitates a multifaceted approach, including enhanced antimicrobial stewardship, better infection control, and improved access to affordable diagnostics and treatments in resource-limited settings.

Molecular techniques for detecting carbapenemase genes, while highly specific, are often expensive, demand considerable expertise, and are constrained by the limited range of targeted genes. In the past decade, various phenotype-based assays have emerged as alternatives. These include growth-based techniques like the modified Hodge test (MHT), Etests, and carbapenem inactivation method (CIM), as well as rapid colorimetric assays like manual and commercial versions of the carba NP test. Furthermore, a variety of techniques for the phenotypic detection of carbapenemase activity are also available, including immunochromatographic tests and matrix-assisted laser desorption/ionization time-of-flight mass spectrometry (MALDI-TOF MS) carbapenem hydrolysis assays [11]. Therefore, this study aims to identify the prevalence of carbapenemase-produ*cing E. coli* and *Klebsiella*, in addition to comparing the effectiveness of two methods for detecting carbapenemase production, with the sensitivity of mCIM being higher than that of MHT, making it more reliable in identifying resistant isolates.

## Materials and methods

Study design, period, and sample size

This study, using a descriptive, cross-sectional design, took place at the Microbiology Department at Krishna Vishwa Vidyapeeth (Deemed To Be University), Karad, Satara, India, from July 2022 to June 2023 after receiving ethical approval from the Institutional Ethics Committee through protocol number 231/2023-2024, and informed consent was taken from all the patients involved.

Inclusion criteria

Non-repetitive midstream urine samples from UTI patients with urine cultures isolating *Klebsiella pneumoniae* and *E.coli* were included.

Exclusion criteria

Isolates from the same patient and specimens will be excluded from the study to avoid duplication of isolates.

Sample collection and processing

Samples from outpatient and inpatient departments were processed using standard microbiology guidelines. Midstream urine was collected aseptically and transported to the laboratory within an hour. The isolates were grown on MacConkey agar and blood agar, identified using Gram stain, and further confirmed with oxidase, catalase, and other biochemical tests. Only *Klebsiella pneumoniae* and *E. coli* were part of this study.

Antimicrobial susceptibility test

Mueller Hinton agar was subjected to antimicrobial susceptibility testing using the Kirby Bauer disc diffusion technique following the Clinical Laboratory Standards Institute (CLSI) recommendations [6]. Isolates with a meropenem inhibition zone diameter of less than 21 mm were positive for carbapenemase screening [12], and further phenotypic confirmatory tests like the modified Hodge test (MHT) and modified carbapenem inactivation method (mCIM) were conducted on these screening-positive isolates to confirm the presence of carbapenemase enzyme.

Modified Hodge test (MHT)

**Figure 1:**
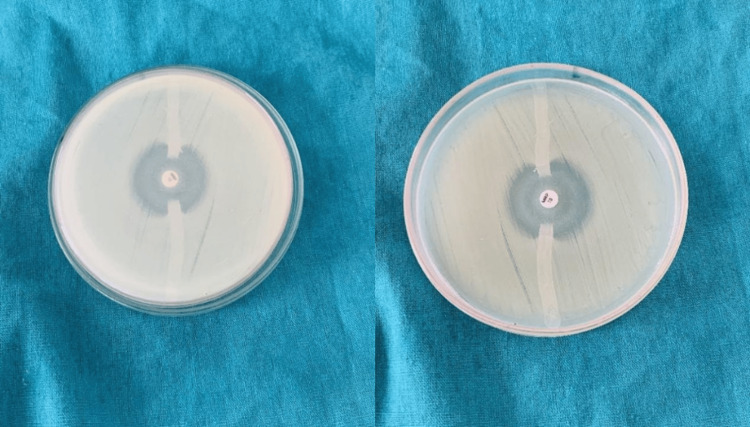
Positive modified Hodge test showing clover-leaf indentation

A 0.5 MacFarland's broth of *E. coli* American Type Culture Collection (ATCC) 25922, diluted 1:10, was used to streak a Mueller Hinton agar plate to make a bacterial lawn, which was then allowed to dry for two to five minutes. A 10 µg Meropenem disc was kept in the center of the plate, and a small amount of zinc sulfate powder was sprinkled onto the disc. The meropenem-resistant isolate was streaked in a straight line from the edge of the disc to the outer edge of the plate. The plate was incubated at 35-37℃ for 16-24 hours. After incubation, the appearance of clover-leaf-shaped indentation (Figure 1) at the junction of ATCC* E.coli* 25922 and the test isolate within the meropenem inhibition zone indicates positive MHT results [12].

Modified carbapenem inactivation method (mCIM)

**Figure 2:**
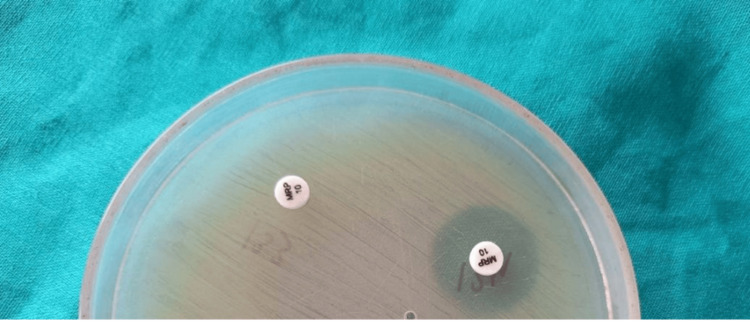
Modified carbapenem inactivation method showing positive result (left meropenem disc) and negative result (right meropenem disc)

A 2 mL of tryptic soy broth was utilized to mix a 1µL loopful of organisms that were cultured overnight on blood agar. Next, the suspension was mixed thoroughly using a vortex for 10-15 seconds. A 10µg meropenem disc was placed within each tube using sterile forceps, making sure the disc was completely immersed in the suspension and incubated for four hours at 35-37℃. Following the incubation period, Mueller Hinton agar plates were made using an ATCC *E. coli* 25922 0.5 MacFarland solution. Following that, the meropenem discs were taken out of the tubes and put on Mueller Hinton agar plates, which were then left to incubate at 37℃ for the entire night. A positive result (Figure 2) is indicated by the inhibition zone size of 6-15mm or the existence of pinpoint colonies in a 16-18mm zone diameter, while the diameter of a zone ≥19mm is considered negative [6].

The MHT has notable limitations in sensitivity and specificity, with sensitivity ranging from 68% to 97% and specificity from 67% to 100%. This variability can result in false negatives, especially in strains with low-level carbapenemase productions, and false positives due to other resistance mechanisms. The mCIM generally demonstrates higher sensitivity, often reaching 100%, but its performance can be influenced by bacterial load and growth conditions. Although mCIM typically maintains high specificity, it may suffer cross-reactivity with other beta-lactamases. Understanding these limitations is essential for accurate diagnosis and emphasizes the importance of additional testing to ensure reliable results.

Statistical analysis 

Data was entered using MS Excel software (Microsoft Corp., Redmond, WA, US), and the chi-square test was subsequently conducted using GraphPad InStat software (Insight Venture Management, LLC, New York, NY, US). A chi-square test was chosen to assess the association between carbapenemase production detection methods (MHT vs. mCIM) and the types of isolates (*Klebsiella* vs. *E. coli*). The analyzed data were then presented as percentages and p-values. A result was deemed statistically significant if the probability was less than 0.05.

## Results

Out of 1,653 clean-catch midstream urine samples from UTI patients that were collected, 150 were *Escherichia coli *and *Klebsiella pneumoniae* each. *E. coli* isolates were most common in the 41-60 year old age group (35%); on the other hand, *Klebsiella pneumoniae* isolates are common in the 41-60 year old age group (40%), as shown in Table 1. This data indicates a higher prevalence of *E. coli* and *Klebsiella pneumoniae *in the 41-60 age group, highlighting a significant demographic for urinary tract infections.

**Table 1:** Age-wise distribution n: number; %: percentage

Age group (in years)	*E. coli*, n(%)	*Klebsiella pneumoniae,* n(%)
0-20	3 (2)	4 (3)
21-40	38 (25)	33 (22)
41-60	52 (35)	60 (40)
61-80	50 (33)	52 (34)
>80	7(5)	1 (1)
Total	150 (100)	150 (100)

*E. coli *had nearly equal gender distribution (49% male, 51% female), while *Klebsiella pneumoniae* had a higher prevalence in males (59%) as depicted by Table 2. *E. coli* isolates show a nearly equal gender distribution, whereas *Klebsiella pneumoniae* is more prevalent in males, suggesting potential gender-related susceptibility or exposure factors.

**Table 2:** Gender-wise distribution n: number; %: percentage

Gender	*E. coli* , n(%)	*Klebsiella pneumoniae*, n(%)
Male	73 (49)	88 (59)
Female	77 (51)	62 (41)
Total	150 (100)	150 (100)

It was observed that the majority of the *E. coli* isolates were from the inpatient department (80%), while the rest were from the outpatient department (20%). Most of the isolates of *Klebsiella pneumoniae* were from the inpatient department (93%), while the rest were from the outpatient department (7%), as seen in Table 3.

**Table 3:** Distribution of isolates among inpatient and outpatient departments n: Number; %: percentage

Organism	Inpatient department, n(%)	Outpatient department, n(%)
E. coli	120 (80)	30 (20)
Klebsiella pneumoniae	140 (93)	10 (7)

The pattern of antibiotic susceptibility revealed that *E. coli* had the highest resistance to ceftazidime (97%) and norfloxacin (97%), and was most sensitive to tigecycline (97%) and fosfomycin (96%). *Klebsiella pneumoniae* showed maximum resistance to ceftazidime (89%) and cefuroxime (89%) and highest sensitivity to tigecycline (86%) and fosfomycin (61%), as shown in Table 4.

**Table 4:** Antibiotic susceptibility profile of E.coli and Klebsiella pneumoniae Chi-square: 1519.7, p-value <0.001, significantly associated. n: number; %: percentage

Antibiotics	E. coli		Klebsiella pneumoniae	
	Sensitive, n(%)	Resistant, n(%)	Sensitive, n(%)	Resistant, n(%)
Amikacin	127 (85)	23 (15)	73 (49)	77 (51)
Cefepime	25 (17)	125 (83)	29 (19)	121 (81)
Cefoxitin	44 (29)	106 (71)	19 (13)	131 (87)
Ceftazidime	4 (3)	146 (97)	16 (11)	134 (89)
Ceftriaxone	16 (11)	134 (89)	18 (12)	132 (88)
Cefuroxime	14 (9)	136 (91)	17 (11)	133 (89)
Ciprofloxacin	7 (5)	143 (95)	23 (15)	127 (85)
Cotrimoxazole	63 (42)	87 (58)	58 (39)	92 (61)
Ertapenem	93 (62)	57 (38)	51 (34)	99 (66)
Fosfomycin	144 (96)	6 (4)	92 (61)	58 (39)
Gentamicin	80 (53)	70 (47)	55 (37)	95 (63)
Imipenem	102 (68)	48 (32)	52 (35)	98 (65)
Meropenem	104 (69)	46 (31)	64 (43)	86 (57)
Nalidixic acid	65 (43)	85 (57)	19 (13)	131 (87)
Nitrofurantoin	57 (38)	93 (62)	54 (36)	96 (64)
Norfloxacin	5 (3)	145 (97)	28 (19)	122 (81)
Tigecycline	146 (97)	4 (3)	129 (86)	21 (14)

Table 5 summarizes the results of the modified Hodge test for *E. coli* and *Klebsiella pneumoniae*. Out of the 150 isolates of *E. coli*, 26 showed a positive result, while 124 were negative. For *Klebsiella pneumoniae*, 46 isolates yielded positive results and 104 were negative. 

**Table 5:** Results of the modified Hodge test Chi-square: 7.310, p-value= 0.0069, significantly associated. n: number

Organism	Positive (n)	Negative (n)
E. coli	26	124
Klebsiella pneumoniae	46	104

Table 6 presents the results of the modified carbapenem inactivation method for *E. coli* and *Klebsiella pneumoniae*. For *E. coli*, 43 isolates were positive, while 107 isolates were negative. In the case of *Klebsiella pneumoniae*, 68 isolates showed positive results, and 82 were negative.

**Table 6:** Results of modified carbapenem inactivation method Chi-square: 8.938, p-value= 0.0028, significantly associated. n: number

Organism	Positive (n)	Negative (n)
E. coli	43	107
Klebsiella pneumoniae	68	82

In the current study, 25 (17%) *E. coli* and 41 (27%) *Klebsiella pneumoniae *tested positive for both MHT and mCIM (Table 7). The results from both MHT and MCIM indicate a substantial proportion of isolates testing positive for carbapenemase production, emphasizing the importance of accurate resistance detection.

**Table 7:** Comparative study of MHT and mCIM for detection of carbapenemase MHT: modified Hodge test; mCIM: modified carbapenem inactivation method; n: number; %: percentage

Organism	MHT POSITIVE n(%)	mCIM POSITIVE n(%)	MHT + mCIM POSITIVE n(%)
E. coli	46 (31)	68 (45)	25 (17)
Klebsiella pneumoniae	26 (17)	43 (29)	41 (27)

These outcomes provide insightful information about the carbapenem resistance mechanisms between *Klebsiella pneumoniae* and *E. coli*, with a majority exhibiting positive reactions in mCIM and a significant proportion also showing positivity in MHT, suggesting the presence of diverse resistance mechanisms in this particular group of isolates. It also emphasizes the significance of utilizing a combination of diagnostic approaches to thoroughly evaluate carbapenem resistance in urinary isolates of *Klebsiella pneumoniae* and *E. coli*.

## Discussion

Gram-negative bacilli cause infections that have emerged as significant challenges for healthcare institutions due to the limited antibiotic options and the associated high mortality rates [13]. As a result, carbapenem resistance has become a critical focal point in the ongoing battle against healthcare-associated infections, demanding increased attention to safeguard patient health and the burden of such infections on healthcare facilities. In a study by Satyajeet K. Pawar et al. [14], 82% of CRE were *Klebsiella pneumoniae* (63%) and *E. coli* (19%), whereas the current study found 44% CRE, with *Klebsiella pneumoniae* accounting for 29% and *E. coli* for 15%.

In the present study, 132 carbapenem-resistant isolates were identified, with 24% positive for MHT and 37% positive for mCIM. This highlights a moderate prevalence of carbapenemase production among isolates tested. In comparison, AP Khare et al. [15] reported 150 carbapenem-resistant isolates, with higher MHT positivity rates of 42% and a similar mCIM positive rate of 42.66%. This suggests a slightly more widespread carbapenemase activity in their study population.

Abed Zahedi et al. [4] reported 122 resistant isolates, with 57.38% positive for MHT and 71.31% on mCIM, reflecting a substantial presence of carbapenemase producers. Amjad et al. [16] identified 200 carbapenem-resistant isolates with a 69% MHT positive rate, which is comparatively high. Lastly, Jayalakshmi et al. [12] reported 152 resistant isolates, with 32.89% positive on MHT.

This study's findings of carbapenem resistance in both *Klebsiella pneumoniae* and *E. coli* are concerning, as carbapenems are crucial antibiotics for treating serious infections. The significant finding of our study, where 24% of the samples tested positive for MHT and 37% positive for mCIM, underscores the concern of high resistance rates and treatment challenges leading to treatment failure. These results also signify the necessity of robust antibiotic stewardship programs within healthcare settings.

Carbapenem resistance shows significant regional variations worldwide. In South Asia, particularly India and Pakistan, rates of CRE exceed 50%, largely due to New Delhi metallo-beta-lactamase (NDM-1) [17]. In a study done in Seoul, Korea, it was observed that *Klebsiella pneumoniae* was the predominant species (56.5% of isolates), followed by *Escherichia coli* (17.0%) as CRE [18]. This aligns with the studies conducted in Bahrain, Taiwan, and the US [19-21], where *K. pneumoniae* was also the most prevalent species, reaching up to 91% in some cases. Given its frequent identification as the leading species among CRE, *K. pneumoniae* is referred to as carbapenem-resistant *K. pneumoniae* and is expected to play a significant role in the spread of carbapenem resistance.

Our study demonstrated that the mCIM method has higher sensitivity compared to the MHT. Saliya Al Musawi’s study revealed the mCIM method demonstrates higher sensitivity compared to MHT for detecting OXA-48 and NDM-type carbapenemases [22]. The mCIM method is well suited for resource-limited microbiological laboratories due to its low cost and simplicity. Additionally, the interpretation of mCIM results is more straightforward and less subjective compared to MHT [23].

## Conclusions

The high prevalence of multidrug-resistant *Klebsiella* and *E. coli* strains, particularly those resistant due to carbapenemase production, poses a significant challenge for treatment and patient outcomes. Accurate detection of carbapenem resistance is crucial, and this study discovered that both the mCIM and MHT are effective diagnostic tools. Importantly, the sensitivity of mCIM was higher than MHT, making it more reliable in identifying resistant isolates and reducing false negatives.

The research additionally highlights the fact that both mCIM and MHT are easy to implement in routine laboratory testing without the need for specialized equipment. This accessibility permits improved integration with standard procedures, aiding clinicians in making informed decisions about antibiotic therapy. In conclusion, the study highlights the prevalence of carbapenemase and the importance of their detection methods in managing drug-resistant infections and improving patient care.
